# Targeted Modulation of Matrix‐Remodeling: Enzymes, Adhesion Receptors, and Signaling Hubs as Precision Strategies for Antifibrotic Therapy

**DOI:** 10.1002/ccs3.70124

**Published:** 2026-11-14

**Authors:** Elke Roeb, Ralf Weiskirchen

**Affiliations:** ^1^ Department of Gastroenterology Justus Liebig University Giessen Giessen Germany; ^2^ Institute of Molecular Pathobiochemistry Experimental Gene Therapy and Clinical Chemistry (IFMPEGKC) RWTH University Hospital Aachen Germany

**Keywords:** fibrocytes, fibrosis, liver, MASLD, matrix, myofibroblast

## Abstract

Liver fibrosis is increasingly recognized as a dynamic, spatially organized, and potentially reversible process of maladaptive wound healing rather than a passive consequence of collagen accumulation. Persistent liver injury activates multicellular fibrotic niches composed of hepatic stellate cells, macrophages, endothelial cells, fibrocytes, circulating myeloid populations, and extracellular matrix components that interact through reciprocal signaling networks. This review outlines a precision‐oriented antifibrotic framework centered on three interconnected target classes: matrix‐remodeling enzymes, adhesion receptors, and intracellular signaling hubs. Matrix metalloproteinases and tissue inhibitors of metalloproteinases regulate extracellular matrix turnover and determine whether fibrotic scars persist, remodel, or regress, indicating that productive fibrolysis should be restored rather than globally enhanced or suppressed. Adhesion receptors, particularly integrins, connect extracellular matrix composition and stiffness to intracellular mechanotransduction, latent TGF‐*β* activation, hepatic stellate cell activation, and fibro‐inflammatory cell recruitment. Signaling hubs such as TGF‐*β*\SMAD, GARP–TGF‐*β*, FAK–RhoA/ROCK, PAK, NF‐*κ*B, and YAP/TAZ integrate mechanical, inflammatory, and stromal cues into persistent profibrotic cellular states. Recent single‐cell, spatial transcriptomic, and spatial multi‐omics studies indicate that fibrosis is driven by disease‐specific cellular neighborhoods rather than uniform activation of single pathways or cell types. These findings support mechanism‐guided strategies that combine molecular target selection with spatial and temporal restriction to preserve physiological repair, immunity, regeneration, and tissue homeostasis. Future antifibrotic therapy will likely depend on identifying the dominant fibrotic niche in individual patients and selectively reprogramming the matrix, cell–matrix interface, and signaling networks that prevent fibrosis resolution.

## Graphical abstract

Liver fibrosis arises from a spatially organized fibrotic niche sustained by reciprocal interactions between extracellular matrix remodeling, cell–matrix adhesion, mechanotransduction, and inflammatory signaling. Emerging antifibrotic therapies aim to restore fibrosis resolution by selectively targeting matrix‐remodeling enzymes, adhesion receptors, and signaling hubs that maintain pathological network activity.

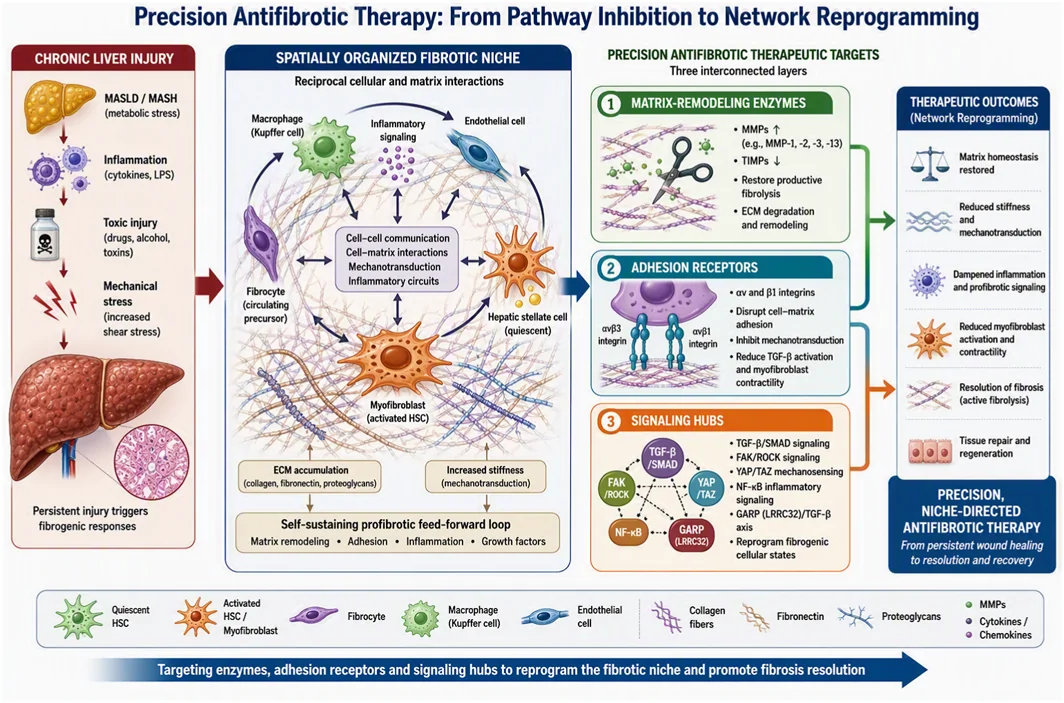

## 1 Introduction

Liver fibrosis is increasingly understood as a dynamic, multicellular process driven by reciprocal interactions between hepatic stellate cells, immune cells, endothelial cells, and the extracellular matrix, rather than as a passive consequence of collagen accumulation [1, 2]. Persistent tissue injury activates a multicellular network comprising hepatic stellate cells (HSCs), macrophages, endothelial cells, fibroblasts, circulating myeloid populations, and extracellular matrix (ECM) [3, 4]. Activated HSCs remain the principal source of collagen‐producing myofibroblasts; however, fibrosis is increasingly understood as an emergent consequence of reciprocal cellular and molecular interactions within a spatially organized fibrotic niche [5, 6, 7]. HSCs are recognized as the principal matrix‐producing cells driving hepatic fibrogenesis, whereas other cell populations, including fibrocytes, may also contribute to extracellular matrix deposition in a context‐dependent manner [8, 9].

This conceptual shift has several important therapeutic implications. Rather than indiscriminately suppressing collagen synthesis or eliminating individual cell populations, future antifibrotic strategies may selectively disrupt the molecular circuits that sustain pathological matrix accumulation, cellular adhesion, and fibro‐inflammatory signaling, while preserving physiological tissue repair [10, 11, 12]. The emerging biology of fibrocytes illustrates this principle. Although fibrocytes represent only a minor fraction of hepatic collagen‐producing cells, experimental depletion can substantially attenuate fibrosis, indicating that their biological impact derives less from matrix production than from their capacity to regulate inflammatory signaling, ECM turnover, and communication with resident stromal cells [9, 12, 13, 14, 15]. In a murine model of thioacetamide‐induced fibrosis, fibrocyte depletion altered IL‐1*β*, LTB4, SMAD6, CEBPB, MMP3, and MMP13, thereby linking fibrocyte biology directly to the inflammatory and matrix‐remodeling pathways [16]. These observations support a precision‐oriented therapeutic framework centered on three interconnected target classes: matrix remodeling enzymes, adhesion receptors, and signaling hubs [17]. Their common feature is that they occupy regulatory positions at the interface between cells and the extracellular environment [18]. Therefore, modulating these nodes may achieve greater specificity than conventional approaches directed against single downstream mediators. This network‐based view of hepatic fibrosis is summarized in Figure 1, which illustrates fibrosis as a spatially organized multicellular niche, rather than a linear consequence of collagen accumulation. The figure also introduces the three target classes that structure this review (i.e., matrix‐remodeling enzymes, adhesion receptors, and signaling hubs), and shows how they converge at the interface between stromal cells, immune cells, endothelial cells, and the ECM.

**FIGURE 1:**
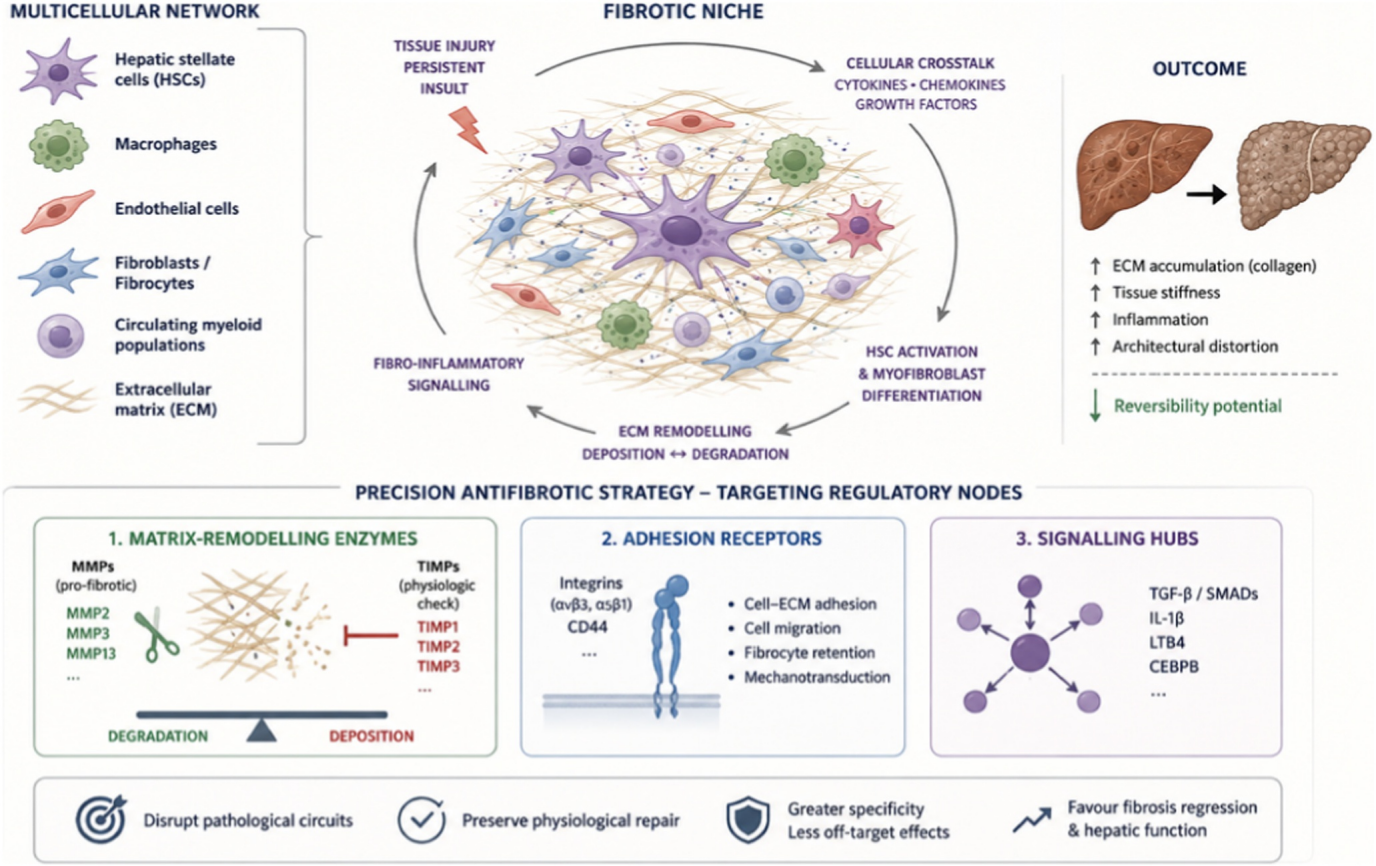
Dynamic liver fibrosis as a spatially organized multicellular niche. This framework provides the rationale for considering the ECM itself as an active therapeutic signaling compartment. Reciprocal cellular signaling and extracellular matrix remodeling is coordinated by matrix‐regulating enzymes, particularly MMPs and TIMPs, alongside adhesion receptors and signaling hubs, thereby sustaining pathological matrix accumulation while offering potential targets for precision antifibrotic therapy.

## 2 The Extracellular Matrix as a Therapeutic Signaling Compartment

The ECM is not merely the end product of fibrogenesis; it is an active signaling compartment that continuously regulates cellular phenotype [19]. Progressive accumulation of fibrillar collagen, altered matrix composition, and increased tissue stiffness enhance integrin‐dependent mechanotransduction, growth‐factor sequestration, and HSC contractility [20, 21, 22]. These changes reinforce myofibroblast activation and perpetuate fibrosis, establishing a self‐sustaining feed‐forward loop [23, 24]. Preclinical in vivo models and three‐dimensional culture systems indicate that increased extracellular matrix (ECM) viscoelasticity can facilitate hepatocellular carcinoma (HCC) cell proliferation and invasion via integrin *β*1–tensin‐1–YAP mechanotransduction. Furthermore, advanced glycation end product (AGE)‐induced alterations in ECM architecture may enhance viscoelastic properties, thereby promoting tumor progression independently of matrix stiffness [25].

This perspective is consistent with single‐cell analyses of the human cirrhotic liver, which have revealed spatially organized scar‐associated macrophages, endothelial cells, and collagen‐producing mesenchymal cells connected through extensive ligand–receptor networks [5]. TNFRSF12A, PDGF, and NOTCH signaling, among others, have been identified as potentially druggable components of this fibrotic niche. Thus, therapeutic intervention should be considered not simply as inhibition of collagen synthesis, but also as reprogramming of the pathological matrisome and its signaling functions [17, 26, 27].

A particularly important consequence is that matrix degradation must be approached precisely. ECM turnover is governed by a tightly regulated balance between matrix metalloproteinases (MMPs), endogenous tissue inhibitors (TIMPs), and other proteolytic systems [4]. Pioneering work has established the central importance of the MMP/TIMP system for both fibrogenesis and fibrolysis and highlighted the therapeutic potential of manipulating matrix turnover rather than merely suppressing matrix synthesis [28, 29, 30]. Therefore, the challenge was not to inhibit MMP activity. Broad‐spectrum MMP inhibition may interfere with physiological matrix turnover, tissue repair, and immune functions. Instead, the selective modulation of individual proteases, protease–substrate interactions, or spatially restricted proteolytic activity may offer a more refined strategy [4, 31, 32]. Figure 2 expands this concept by depicting ECM remodeling as a dynamic equilibrium between matrix deposition, proteolytic degradation, and mechanotransductive feedback. By emphasizing the balance between MMP activity and TIMP‐mediated inhibition, this figure highlights why therapeutic strategies should aim to restore productive fibrolysis rather than globally enhance or suppress protease activity.

**FIGURE 2:**
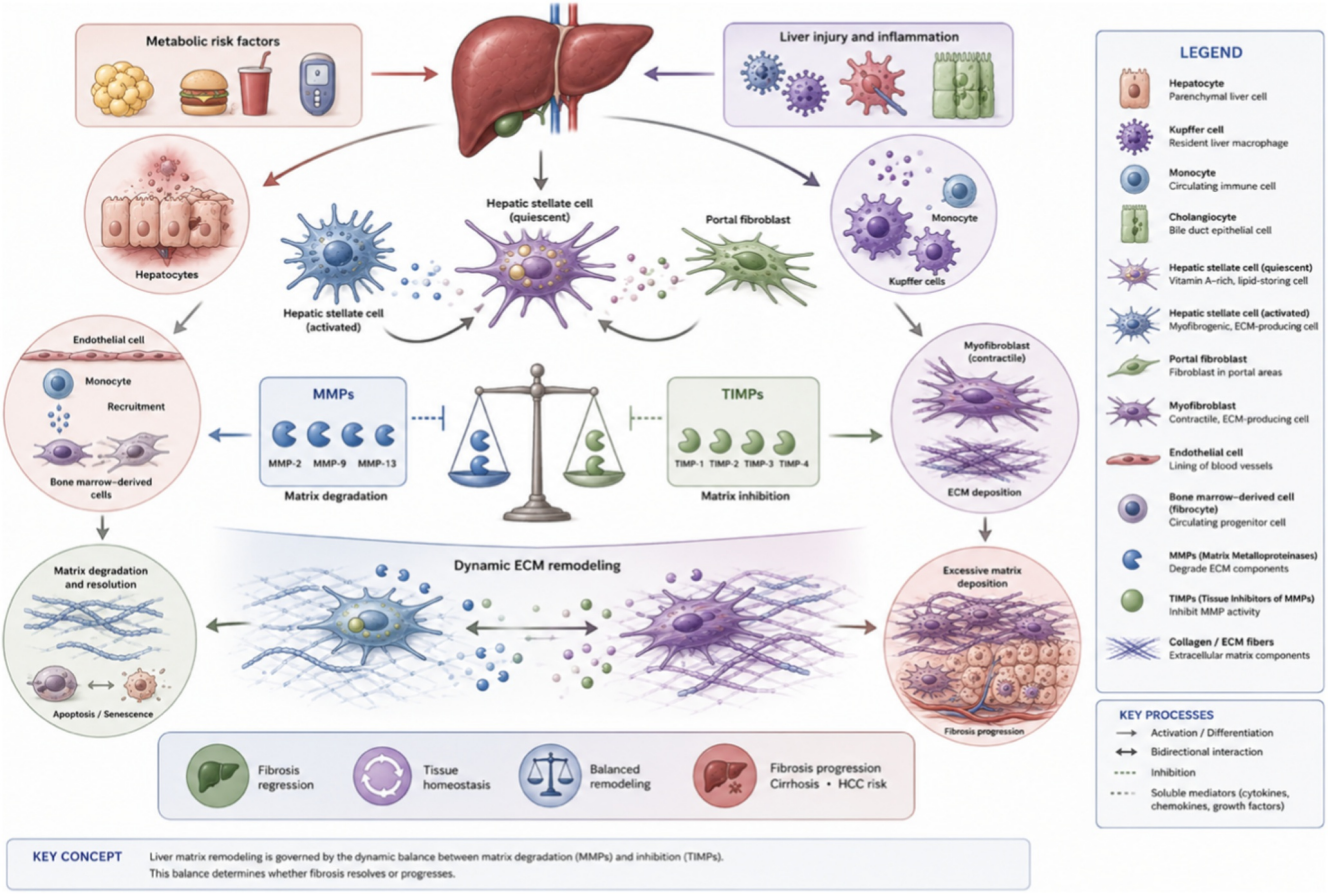
Dynamic extracellular matrix remodeling in the liver. This dynamic view of matrix turnover provides the conceptual basis for targeting matrix‐remodeling enzymes as functional regulators of fibrosis regression. Liver injury and metabolic or inflammatory stress activate a multicellular network involving hepatocytes, Kupffer cells, monocytes, cholangiocytes, portal fibroblasts, and hepatic stellate cells (HSCs). Quiescent HSCs undergo activation and differentiation into contractile, matrix‐producing myofibroblasts, accompanied by extensive changes in the extracellular matrix (ECM). Matrix turnover is dynamically regulated by the balance between matrix‐degrading matrix metalloproteinases (MMPs) and their endogenous inhibitors, tissue inhibitors of metalloproteinases (TIMPs). Increased MMP activity promotes ECM degradation and remodeling, whereas sustained TIMP activity limits proteolysis and favors matrix accumulation. The resulting equilibrium between matrix deposition, degradation, and cellular mechanotransduction determines whether fibrosis resolves or progresses. Thus, hepatic fibrosis is not a static process of collagen accumulation, but a dynamic and potentially reversible state of continuous ECM remodeling.

## 3 Matrix‐Remodeling Enzymes: Restoring Productive Fibrolysis

MMPs occupy a particularly attractive position because they determine not only the quantity, but also the quality and signaling activity of the ECM. Proteolytic cleavage can remove pathological matrix components, release matrix‐bound growth factors, and generate bioactive matrikines [33]. Fibrocytes express several MMPs and TIMPs, and may therefore influence matrix architecture and signaling without substantially contributing to the total collagen burden [9, 12]. Our previous work was instrumental in establishing this concept. Our studies demonstrated that MMPs and TIMPs are dynamically regulated during hepatic fibrogenesis and regression, and experimental modulation of this system can influence fibrosis [16, 29, 31, 34]. Importantly, subsequent work has shown that altered MMP activity can contribute to antifibrotic effects in vivo [31]. In *Abcb4*‐deficient mice, bone marrow transplantation improved hepatic fibrosis in association with altered matrix metalloproteinase activity, providing further evidence that matrix degradation is mechanistically linked to fibrosis resolution [34].

Recently, fibrocyte depletion has provided additional mechanistic clues. Loss of fibrocytes was accompanied by increased MMP‐13, together with changes in MMP‐3, suggesting that fibrocytes may maintain fibrosis partly by regulating the balance between matrix deposition and degradation [9]. The increase in MMP‐13 levels following fibrocyte depletion is particularly intriguing because enhanced collagenolytic activity may facilitate scar remodeling and resolution. These findings remain exploratory, but they identify MMP‐13 and related proteolytic pathways as candidate functional nodes rather than merely as biomarkers of fibrosis [30]. Therefore, the therapeutic objective should be to restore productive fibrolysis rather than simply increasing protease activity [35]. Potential strategies include the selective activation of matrix‐degrading enzymes, inhibition of pathologically dominant TIMPs, interference with specific protease–substrate interactions, or spatially targeted delivery to activated stromal niches. The underlying principle is that a fibrotic liver should not simply produce less matrix; it should regain the capacity to remodel and remove the pathological matrix [36]. This distinction is particularly relevant because regression of fibrosis is biologically feasible. Following the removal of the underlying injury, activated myofibroblasts can disappear, and the scar can undergo resorption. Understanding these endogenous mechanisms of regression provides a conceptual basis for therapies designed to accelerate rather than merely prevent fibrosis [22].

Beyond matrix stiffness, liver fibrosis is shaped by a complex interplay of cellular plasticity, mechanotransduction and extracellular matrix (ECM) dynamics [35]. Although hepatic stellate cells (HSCs) are considered the predominant source of matrix‐producing cells in most settings, the contribution of fibrocytes remains controversial and context‐dependent [14, 35]. ECM remodeling affects not only tissue stiffness but also viscoelastic properties, which may independently influence cellular behavior and disease progression [27]. These processes are regulated by interconnected signaling networks extending beyond the pathways discussed here. Moreover, the ECM serves as a reservoir for bioactive mediators, including hepatocyte growth factor (HGF), linking matrix remodeling to tissue repair and liver regeneration [37].

## 4 Adhesion Receptors: Targeting the Interface Between Cells and Matrix

The second class of precision targets comprises adhesion receptors, particularly integrins. These molecules provide a direct molecular interface between ECM and intracellular signaling pathways [10, 26, 38, 39]. Their therapeutic importance is derived from their dual function: they regulate cellular adhesion and migration while simultaneously converting the mechanical and biochemical properties of the matrix into intracellular signals [10, 22, 26]. Among these receptors, *α*v‐containing integrins have emerged as particularly compelling antifibrotic targets because they can activate latent TGF‐*β* [40, 41]. Genetic depletion of αv integrin in HSC‐derived myofibroblasts protects against experimental liver fibrosis, whereas pharmacological inhibition attenuates fibrosis in therapeutic settings [42, 43]. These findings established *α*v integrin signaling as a core pathway linking the extracellular matrix to TGF‐*β* activation and fibrogenesis [44, 45]. The importance of integrin signaling extends beyond *α*v‐mediated TGF‐*β* activation. Integrin *β*1 signaling in hepatic myofibroblasts has been linked to PAK and YAP1, which integrate matrix adhesion, mechanotransduction, cell contraction, and fibrogenic gene expression [22, 46]. Pharmacological inhibition of these downstream pathways attenuates liver fibrosis in vivo. Thus, integrins can be viewed as molecular transducers that connect matrix composition and stiffness to intracellular profibrotic programs [22, 26].

This is particularly relevant to fibrocyte biology. Fibrocyte recruitment involves endothelial adhesion molecules, such as ICAM‐1 and VCAM‐1, together with integrin‐mediated interactions with the activated hepatic endothelium [26]. Once within the tissue, fibrocytes encounter altered ECM enriched in fibronectin, hyaluronan, collagen‐derived matrikines, and other adhesive cues. Therefore, chemokine signaling and matrix‐mediated adhesion cooperate to determine fibrocyte localization and persistence within fibro‐inflammatory niches. Consequently, there are two therapeutic opportunities. First, adhesion receptors can be targeted to limit the pathological recruitment and retention of circulating fibroinflammatory cells. Second, they can be targeted to interrupt matrix‐dependent activation of resident HSCs and fibrocytes after tissue entry [22]. Importantly, broad inhibition of leukocyte trafficking is unlikely to be sufficient. The chemokine and adhesion networks are highly redundant and participate in physiological immune surveillance and repair. The failure of broad CCR2‐directed approaches to consistently translate into effective anti‐fibrotic therapies illustrates this problem. Precision strategies should, therefore, seek to identify disease‐ and niche‐specific adhesion programs rather than globally suppressing immune cell recruitment [47, 48].

## 5 Signaling Hubs: From Individual Cytokines to Network Control

The third, and perhaps most powerful, therapeutic opportunity lies in signaling hubs. Fibrogenesis is driven by interconnected pathways rather than isolated cytokines. TGF‐*β*, PDGF, NF‐*κ*B, Hippo‐YAP/TAZ, PI3K–AKT, NOTCH, and other pathways converge on a limited number of cellular phenotypes, including myofibroblast activation, matrix production, contractility, survival, migration, and inflammatory amplification [49]. The central role of TGF‐*β* remains undisputed, but direct systemic inhibition is problematic because TGF‐*β* has essential physiological functions in immune regulation, tissue repair, and homeostasis [16, 50, 51]. Therefore, more selective approaches aim to interfere with TGF‐*β* activation or cell‐specific signaling. Integrins represent one such upstream mechanism, whereas GARP represents another [40]. Recent work has demonstrated that GARP on HSCs functions as a latent TGF‐*β* docking molecule and contributes to fibrosis through TGF‐*β* activation, identifying GARP as a potentially more selective target than global TGF‐*β* inhibition [40].

Similarly, fibrocyte depletion experiments performed by Hempel et al. identified SMAD6 as a potentially important regulatory node [9]. SMAD6 integrates TGF‐*β*/BMP and inflammatory signaling, including NF‐*κ*B‐related pathways, and its alteration following fibrocyte depletion suggests that fibrocytes may influence fibrosis through the modulation of intracellular signaling rather than simply through matrix production [52, 53].

The importance of such hubs is that they provide leverage; the modulation of one highly connected node may simultaneously alter several pathological programs. However, this also poses a major safety challenge. Ideally, the optimal therapeutic target should be context‐dependent, activated predominantly in the fibrotic niche, and dispensable for normal tissue function [5]. The discovery of scar‐associated cellular states provides a route toward this objective. Single‐cell and spatial analyses have demonstrated that fibrogenesis occurs in discrete cellular niches rather than uniformly throughout the liver. Consequently, the future of antifibrotic therapy may lie in targeting signaling hubs within defined cellular states or anatomical niches rather than inhibiting the same pathway throughout the organism [54, 55, 56, 57].

### 5.1 Spatially Restricted Fibrotic Niches as Therapeutic Units

Recent advances in spatial transcriptomics have fundamentally altered the way fibrosis is conceptualized. Rather than representing a homogeneous accumulation of activated myofibroblasts throughout the liver, fibrosis is increasingly recognized as a spatially organized process in which distinct populations of hepatocytes, endothelial cells, macrophages, and mesenchymal cells form discrete fibroinflammatory niches. A 2025 single‐cell spatial map of healthy and fibrotic human livers identified distinct HSC and macrophage populations and demonstrated substantial remodeling of their spatial relationships during chronic injury [58]. This spatial perspective is particularly relevant to therapeutic targeting. A molecular pathway may be highly active within a discrete fibrotic niche while remaining largely dispensable in healthy tissues. Consequently, therapeutic specificity may be achieved not only by selecting the appropriate molecular target but also by restricting its modulation to the anatomical and cellular context in which it drives disease [17, 59].

This concept has been reinforced by a recent spatial multi‐omics analysis of 61 human livers across the spectrum from metabolic dysfunction‐associated steatotic liver disease (MASLD) to the more progressive form of metabolic dysfunction‐associated steatohepatitis (MASH) [60]. This study integrated single‐cell transcriptomics, spatial transcriptomics, and spatial metabolomics and identified a fibrosis‐associated program enriched in advanced MASH, including spatially restricted crosstalk between central vein endothelial cells and hepatic stellate cells. These findings suggest that the fibrotic phenotype is generated by localized cell–cell communication rather than by uniform activation of individual cell types [60].

This distinction is therapeutically important. If central vein endothelial cells, HSCs, and macrophages form a spatially defined signaling unit, targeting one component of this network may be more effective than systemic inhibition of a ubiquitous cytokine [58, 61]. Equally, this raises the possibility that therapeutic delivery systems can exploit receptors selectively enriched in activated stromal or endothelial niches to concentrate antifibrotic agents at the site of pathological activity [62, 63].

These implications extend to the fibrocytes. Their abundance in fibrotic livers may be relatively modest, but their localization within fibro‐inflammatory niches could allow them to exert disproportionate effects through paracrine signaling and matrix remodeling [9, 12, 64]. Their phenotypic overlap with inflammatory monocytes remains a major obstacle to their identification in human spatial atlases [65], yet this plasticity reinforces the concept that functional states rather than rigid cellular identities may constitute more appropriate therapeutic units [27].

### 5.2 Integrin–YAP Mechanotransduction: Targeting the Fibrotic Feedback Loop

One of the most compelling examples of a therapeutically actionable signaling circuit is the integrin–mechanotransduction axis [22]. Progressive ECM accumulation increases tissue stiffness, which enhances integrin engagement and focal adhesion formation. This activates FAK, RhoA–ROCK, and actomyosin contractility, promotes nuclear YAP/TAZ localization, and reinforces the expression of profibrotic genes. Simultaneously, *α*v integrins facilitate the activation of latent TGF‐*β* [23, 66]. Thus, matrix deposition is converted into intracellular signaling that drives further matrix production through a self‐reinforcing mechanochemical feedback loop [22].

This mechanism provides an important rationale for moving beyond the direct integrin blockade. As integrins are widely expressed and participate in physiological adhesion and tissue repair, systemic inhibition may have undesirable consequences. Targeting downstream effectors such as FAK, ROCK, PAK, or YAP/TAZ may provide a wider therapeutic window. Indeed, earlier work has demonstrated that *β*1‐integrin signaling in hepatic myofibroblasts is coupled to PAK and YAP1, and that pharmacological inhibition of these downstream pathways attenuates experimental liver fibrosis [43, 46].

Recent evidence of congestive hepatopathy has strengthened the translational importance of this pathway. A study in *Gastroenterology* from 2026 demonstrated that hydrostatic pressure activates YAP through integrin *α*V in liver sinusoidal endothelial cells, inducing CCN2/CTGF and collagen IV expression. Endothelial CCN2/CTGF promotes collagen I and IV production by HSCs [44]. Both endothelial CCN2/CTGF deletion and *α*V integrin inhibition reduced fibrosis and portal hypertension, whereas CCN2/CTGF deletion additionally suppressed liver tumorigenesis. Importantly, spatial transcriptomics of human Fontan‐associated liver disease reproduced pericentral activation of the integrin *α*V–YAP–CCN2/CTGF axis [44]. This study is particularly instructive because it demonstrated that the same molecular pathway can connect mechanical stress, endothelial dysfunction, matrix remodeling, HSC activation, and carcinogenesis within a defined anatomical niche. Therefore, it provides a compelling proof‐of‐principle for precision antifibrotic therapy directed at a mechanosensitive signaling hub rather than at collagen synthesis [10].

However, its therapeutic implications are still broader. The integrin–FAK–RhoA/ROCK–YAP network represents a convergence point for matrix composition, mechanical stiffness, and soluble profibrotic signals. The selective modulation of this network can interrupt several reinforcing mechanisms simultaneously. Conversely, because YAP and its related pathways also regulate regeneration and tissue homeostasis, therapeutic intervention will probably need to be temporally and spatially restricted [27, 44].

### 5.3 Heterogeneity of Hepatic Stellate Cells: Why Indiscriminate Targeting May be Counterproductive

An important refinement of the HSC paradigm has emerged in recent single‐cell and spatial studies. HSCs are not a homogeneous population of collagen‐producing cells, but comprise spatially and functionally distinct states with divergent roles in homeostasis, regeneration, and fibrosis [59, 67, 68]. A comprehensive 2025 review in *Nature Reviews Gastroenterology & Hepatology* emphasized that HSCs perform important physiological functions extending beyond fibrogenesis, including the regulation of hepatic zonation, metabolism, injury responses, regeneration, and the identity of other nonparenchymal cells [59]. This recognition has major implications for antifibrotic drug development; the therapeutic objective should be to selectively reprogram pathogenic HSC states rather than to eliminate or globally suppress HSCs [59]. Spatial studies have supported this distinction. Recent studies identified distinct portal and central HSC populations with distinct ECM and receptor programs. In experimental fibrosis, central/perivenous HSCs appear particularly prone to differentiate into collagen‐producing myofibroblasts, whereas portal HSCs may adopt alternative phenotypes [69].

This heterogeneity provides a conceptual framework for precision targeting [62, 70]. Rather than asking which molecule activates HSCs in general, the more relevant question is: Which molecular circuit defines the pathogenic HSC state in a particular disease and anatomical compartment? Such an approach may reveal selective dependence on integrin–YAP signaling, PDGF signaling, TGF‐*β* activation, matrix proteolysis, or specific transcriptional regulators [70]. The same principle applies to fibrotic regression [11, 19, 35]. Not all activated HSCs necessarily have the same capacity to revert, undergo apoptosis, or adopt alternative states after the removal of the injurious stimulus. Therapeutic strategies that promote the transition from pathogenic myofibroblast states toward quiescent or restorative states may therefore prove superior to approaches based solely on cell depletion [71]. This is especially relevant in the context of MASLD/MASH, in which metabolic injury, inflammation, and fibrosis coexist, but are not biologically synonymous. Therefore, a therapy directed against a fibrosis‐associated HSC program may complement, rather than replace, interventions targeting the metabolic and inflammatory drivers of the disease [72].

### 5.4 Matrix Proteolysis as a Spatially Controlled Therapeutic Process

The concept of matrix remodeling also warrants further refinement. The traditional view of MMPs as either profibrotic or antifibrotic is becoming increasingly inadequate. MMPs can degrade structural matrix components but can also release growth factors, generate matrikines, and modify cell migration and inflammatory signaling. Consequently, the biological effect of an individual MMP depends on its substrate repertoire, cellular source, subcellular localization, and the stage of fibrosis [26, 34, 39, 73, 74].

This complexity provides a rationale for selective rather than global modulation of proteolysis. Our work has long emphasized the importance of MMP/TIMP balance in liver fibrosis and regression [4, 75]. More recent studies further support the concept that matrix proteolysis should be regarded as an active regulatory process rather than a simple mechanism for collagen removal [39, 76, 77, 78]. Fibrocyte data adds an intriguing dimension to this concept. Fibrocyte depletion was associated with changes in MMP3, MMP13, IL‐1*β*, LTB4, SMAD6, and CEBPB. These observations suggest that fibrocytes may regulate matrix turnover indirectly by controlling the inflammatory and transcriptional programs that determine protease expression [9].

Thus, rather than developing conventional broad‐spectrum MMP inhibitors or nonspecific protease activators, future approaches might exploit protease–substrate specificity, cell‐selective delivery, or local activation. The objective is to restore the architecture of the ECM without disrupting physiological matrix turnover elsewhere [4]. This approach is consistent with the broader evolution of ECM biology. Modern matrisome research increasingly views the ECM as a dynamic signaling reservoir, whose composition, post‐translational modification, and proteolytic processing influence cellular identity. Naba recently highlighted the complexity of ECM assembly and remodeling, and the functional importance of minor matrix constituents beyond the major structural collagens [17]. For antifibrotic therapy, this implies that matrix quality may be at least as important as matrix quantity [18, 26, 28]. A therapy capable of selectively removing pathological collagen cross‐links, altering matricellular proteins, or restoring productive proteolysis could potentially normalize the fibrotic niche, even without completely eliminating scar tissue.

### 5.5 Common and Organ‐Specific Fibrotic Niches: Lessons From Liver Research

Across lung, kidney and heart, fibrotic niches share a core mechanism: persistent injury acti vates stromal cells, disrupts matrix metalloproteinase/tissue inhibitor of metalloproteinase (MMP/TIMP) balance and promotes extracellular matrix (ECM) accumulation [79]. Integrins sense matrix composition and stiffness, activating focal adhesion kinase and YAP/TAZ mechanotransduction, which reinforce myofibroblast activation and matrix deposition [80]. However, organ‐specific architecture and mechanical demands shape these responses. In the lung, alveolar epithelial injury and aberrant repair dominate; in the kidney, tubular damage, pericyte‐derived stromal cells and interstitial matrix expansion drive progression; in the heart, mechanical loading, cardiomyocyte injury and conduction‐disrupting interstitial or replacement fibrosis are central [81]. Fibrocytes, circulating bone‐marrow‐derived cells with fibroblast‐like features, may contribute to inflammation and matrix remodeling, although their quantitative contribution varies by organ and remains debated [82]. Liver research provides a translational framework: hepatic stellate‐cell activation, matrix turnover and stiffness‐dependent signaling offer mechanistic models for identifying conserved fibrogenic pathways [7]. Cross‐organ validation using spatial profiling, lineage‐aware cell analysis and functional perturbation should distinguish shared therapeutic targets from organ‐specific vulnerabilities [79, 83].

### 5.6 From Target Discovery to Mechanism‐Guided Combination Therapy

The convergence of these findings suggests a new model for the development of anti‐fibrotic drugs. The relevant therapeutic unit is neither a single cytokine nor a single cell but a pathological signaling module embedded within a spatially defined niche. Three layers of intervention can be envisaged.

1. Matrix layer—restore ECM turnover.
  Selectively modulates MMPs, TIMPs, collagen processing, cross‐linking, and matricellular proteins to promote productive fibrolysis.
2. Interface layer—disrupt pathological cell–matrix communication.
  Target integrins, focal adhesions, and adhesion receptors couple ECM composition and stiffness with cellular activation.
3. Signaling layer—reprogram intracellular state.
  Modulates TGF‐*β*/SMAD, YAP/TAZ, PAK, FAK, ROCK, NF‐*κ*B, or related signaling hubs that integrate mechanical and inflammatory inputs. Other key signaling pathways have been comprehensively reviewed elsewhere and are therefore not discussed in detail here [35, 84].

The advantage of this architecture is that it accommodates the biological redundancy of the fibrosis. Rather than attempting to completely suppress one pathway, combination therapy can intervene at complementary levels of the same pathological feedback loop. For example, metabolic disease modification could remove the initiating injury, whereas integrin–YAP modulation interrupts mechanotransduction, and selective enhancement of matrix turnover promotes scar resolution [11, 22].

Such combinations may ultimately be tailored to the dominant molecular phenotypes of the individual patients. Spatial transcriptomics, proteomics, and ECM profiling can identify whether fibrosis is primarily characterized by inflammatory recruitment, excessive matrix deposition, impaired fibrolysis, mechanotransduction, or a combination thereof [55, 58, 60, 76]. Functional perturbation can then establish the dominant node. Recent spatial multiomics studies have demonstrated that this level of molecular stratification is becoming technically feasible. The 2025 human MASLD atlas [60], for example, integrated more than 500,000 single cells with spatial transcriptomics and spatial metabolomics and identified fibrosis‐associated endothelial–HSC interactions specifically enriched in advanced disease. Therefore, the logical next step is functional spatial precision medicine: identifying a molecularly defined niche, perturbing its candidate signaling nodes experimentally, and then translating the resulting signature into a biomarker‐guided therapeutic strategy. Taken together, recent developments suggest that antifibrotic therapy is entering a phase in which the mechanism, location, and cellular state may become as important as the fibrosis stage. The transition from isolated molecular targets to spatially organized therapeutic modules is shown in Figure 3. The figure contrasts the traditional linear model of liver fibrosis with an emerging network model in which inflammatory cues, mechanical forces, matrix remodeling, cell–matrix adhesion, and intracellular signaling amplification form a self‐sustaining fibrotic circuit.

**FIGURE 3:**
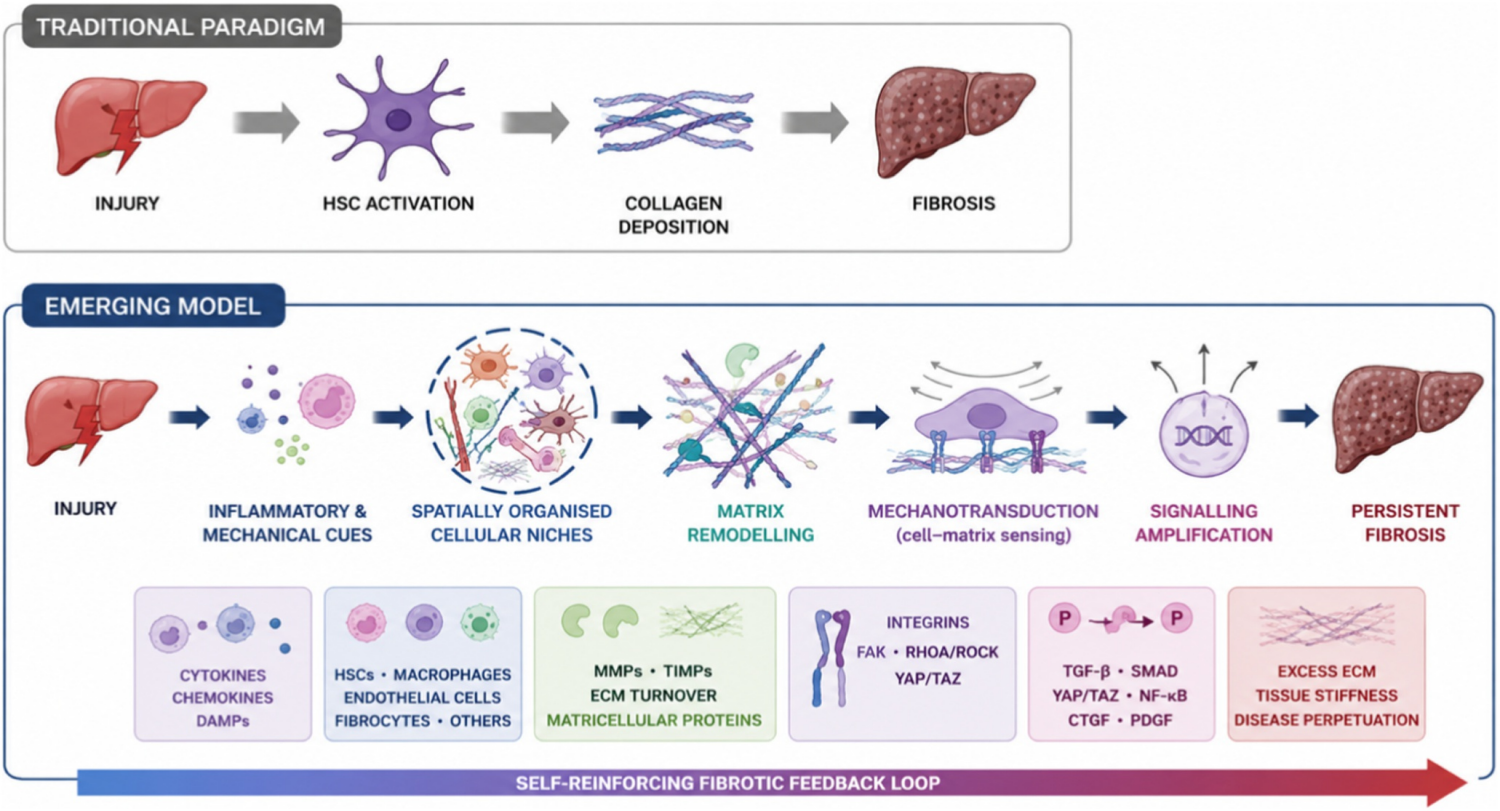
Modern model of hepatic fibrosis. From a linear to a dynamic model of hepatic fibrosis. The traditional paradigm depicts fibrosis as a linear sequence from tissue injury through HSC activation and collagen deposition to fibrosis. The emerging model highlights the interplay of inflammatory and mechanical cues, spatially organized cellular niches, matrix remodeling, mechanotransduction and signaling amplification, forming a self‐reinforcing network that sustains fibrosis.

This network model directly leads to the central translational question addressed in the final section: how can we identify which molecular circuit is dominant in an individual patient's fibrotic niche?

### 5.7 Therapeutic Delivery, Safety, and Spatial Restriction

A central challenge for precision antifibrotic therapy is that many of the most attractive molecular targets are not fibrosis specific. MMPs, TIMPs, integrins, TGF‐*β*‐related pathways, FAK, ROCK, and YAP/TAZ participate in physiological repair, immune regulation, vascular homeostasis, and tissue regeneration [22, 77, 78]. Therefore, the therapeutic problem involves not only target selection but also the control of dose, timing, cellular specificity, and anatomical distribution.

This is particularly relevant for approaches directed toward matrix proteolysis. Excessive or incorrectly timed protease activation can destabilize tissue architecture, release profibrotic or proinflammatory matrikines, or interfere with normal wound healing. Conversely, broad inhibition of proteases may impair physiological ECM turnover and delay resolution of fibrosis. Therefore, future strategies should prioritize local or conditional modulation of proteolytic activity, for example, through prodrugs activated in the fibrotic microenvironment, ligand‐directed nanoparticles, antibody‐drug conjugates, or delivery systems targeted to activated HSCs, liver sinusoidal endothelial cells, or scar‐associated macrophage populations [78]. Similar considerations apply to the adhesion receptors and mechanotransduction pathways. Integrins are attractive because they connect matrix composition, stiffness, latent TGF‐*β* activation, and intracellular signaling; however, they are also widely required for normal cell adhesion and tissue integrity. Rather than attempting complete systemic blockade, therapeutic strategies may need to focus on disease‐enriched integrin combinations, downstream mechanotransduction nodes, or transient inhibition during phases of active fibrogenesis. Spatially resolved molecular profiling could help identify cell populations that express the relevant receptors in a disease‐specific context and thereby guide safer therapeutic delivery.

The same principle is relevant for signaling hubs, such as TGF‐*β*/SMAD, NF‐*κ*B, FAK–RhoA/ROCK, and YAP/TAZ [66]. These pathways provide therapeutic leverage because they integrate multiple profibrotic inputs; however, this leverage also increases the risk of unintended effects on immunity, regeneration, and tumor surveillance [20, 66, 78]. Precision antifibrotic therapy will therefore require not only molecular specificity, but also spatial and temporal restrictions. In practical terms, the most promising interventions may be those that selectively reprogram an activated fibrotic niche while preserving the homeostatic functions of the same pathways in healthy liver tissue [5, 78].

## 6 From Molecular Targets to Precision Antifibrotic Therapy

Antifibrotic drug development increasingly targets extracellular matrix remodeling and mechanotransduction. Integrin inhibition is a promising strategy: the dual *α*v*β*6/*α*v*β*1 inhibitor bexotegrast showed encouraging exploratory signals in the phase 2a INTEGRIS‐IPF trial, although these findings require confirmation in adequately powered trials [85]. ROCK signaling represents another potential target linking cytoskeletal tension, fibroblast activation and matrix deposition; recent preclinical and early clinical findings with selective ROCK2 inhibition in liver fibrosis support further investigation [86]. By contrast, broad‐spectrum matrix metalloproteinase (MMP) inhibition illustrates the risks of insufficient pathway specificity: MMPs regulate not only matrix degradation but also growth‐factor bioavailability, inflammation and tissue repair. Their inhibition may therefore produce context‐dependent effects rather than uniformly suppressing fibrosis. More generally, disappointing clinical translation of several mechanistically plausible antifibrotic candidates highlights the limitations of extrapolating from experimental models to heterogeneous human disease [49, 79].

### 6.1 Major Challenges

The major challenge is to determine which patients harbor a therapeutically actionable fibrotic niche. Fibrosis is biologically heterogeneous; patients with similar fibrosis stages may differ substantially in inflammatory activity, matrix composition, cellular organization, and dominant signaling pathways. Consequently, a single anti‐fibrotic mechanism is unlikely to be equally effective in all patients [1, 11, 67, 87]. Spatial biology is an important solution for this problem. Single‐cell and spatial transcriptomics, spatial proteomics, multiplex imaging, and computational ligand–receptor analysis can identify the cellular neighborhoods in which particular signaling circuits are active. Such approaches could enable the development of molecularly stratified antifibrotic therapies, in which patients are selected according to the dominant mechanism driving their fibrosis.

For example, a patient with a stiff, integrin‐rich matrix niche might preferentially benefit from an adhesion‐ or mechanotransduction‐directed intervention, a patient with excessive matrix accumulation and impaired fibrolysis might be better suited to selective modulation of MMP/TIMP activity, and a patient characterized by strong inflammatory–stromal communication might benefit from targeting a highly connected signaling hub [20, 22, 24, 31, 51, 88]. This approach also favors rational combinations. Because fibrosis is sustained by redundant and compensatory pathways, simultaneous modulation of complementary nodes may be more effective than the complete inhibition of a single pathway. Therefore, a rational combination could pair metabolic disease modification with the selective inhibition of pathological integrin signaling, enhancement of matrix resolution, or modulation of fibro‐inflammatory signaling. The objective would not be maximal pathway suppression but the restoration of network homeostasis.

The translation of niche‐directed antifibrotic therapy requires biomarkers that identify the dominant fibrotic mechanism in individual patients. Conventional fibrosis staging captures the amount and architectural distribution of scar tissue; however, it does not necessarily reveal whether fibrosis is driven by inflammatory recruitment, impaired matrix degradation, pathological mechanotransduction, endothelial–HSC crosstalk, or persistent TGF‐*β* activation. This distinction is critical because therapies directed at MMP/TIMP balance, integrin signaling, or intracellular signaling hubs are unlikely to be equally effective in all molecular fibrosis states [89, 90].

### 6.2 Biomarker‐Guided Stratification: From Clinical Application to Spatial Precision

Biomarkers for fibrosis can be broadly classified into three categories according to their clinical maturity. First, established noninvasive tests such as the FIB‐4 index and vibration‐controlled transient elastography (VCTE), are available for clinical risk stratification and the assessment of liver fibrosis, although their diagnostic performance depends on the underlying disease and clinical context [91]. Second, circulating biomarkers reflecting extracellular matrix turnover, including markers of collagen formation and degradation, offer potential for more dynamic assessment of fibrogenic activity, but their clinical utility and validation vary [92, 93, 94]. Third, tissue‐based molecular profiling and spatial multi‐omics approaches can resolve cell‐specific programs, intercellular interactions and distinct fibrotic niches, thereby providing mechanistic insights and identifying potential therapeutic targets [5, 58, 95]. However, these approaches remain largely investigational and are not yet suitable for large‐scale routine clinical implementation. Key barriers include analytical standardization, independent clinical validation, assay costs, turnaround times and integration into established diagnostic workflows [95, 96]. A stepwise translational strategy linking clinically accessible noninvasive tests with validated molecular signatures may ultimately enable more precise patient stratification and the development of niche‐directed antifibrotic therapies [5, 91, 95].

A practical biomarker strategy can combine histological, molecular, imaging, and circulating readouts. Tissue‐based approaches include spatial transcriptomics, multiplex imaging, ECM proteomics, and ligand–receptor analysis to identify the active fibrotic niche [58, 90]. Circulating markers include collagen formation and degradation products, TIMP‐related signatures, inflammatory mediators, and markers of endothelial and stromal activation. Imaging‐based approaches, including elastography and emerging molecular imaging tools, can help capture matrix stiffness, disease distribution, and longitudinal treatment responses [97, 98].

These biomarkers should be linked to the mechanism‐specific trial endpoints. For a therapy designed to restore fibrolysis, the relevant endpoints may include changes in collagen degradation products, MMP/TIMP‐associated signatures, scar architecture, and quantitative ECM remodeling. For an intervention targeting integrin–YAP mechanotransduction, endpoints may include changes in stiffness, YAP/TAZ target gene expression, focal adhesion signatures, and HSC activation markers. For therapies aimed at inflammatory–stromal crosstalk, endpoints may include macrophage–HSC interaction signatures, chemokine networks, and spatial redistribution of scar‐associated immune cells [58, 89].

Such a framework would allow clinical trials to move beyond unselected antifibrotic treatments and toward mechanism‐guided enrichment. Patients can be selected according to the presence of a defined matrix, interface, or signaling‐layer abnormality, and pharmacodynamic biomarkers can confirm whether the intended niche has been modulated [89, 90]. This approach may be especially important in MASLD/MASH, where metabolic stress, inflammation, hepatocyte injury, and fibrosis may coexist but differ in their relative contributions to disease progression. Ultimately, successful antifibrotic drug development may depend as much on identifying the right patients and fibrotic niches to identify the right molecular target [89, 95].

Taken together, emerging evidence suggests a shift from a cell‐centered to a network‐centered antifibrotic strategy. Three principles may define the next generation of therapy:

1. Remodeling of the matrix rather than merely suppressing its production: Selective manipulation of MMPs, TIMPs, collagen cross‐linking, and matrix‐associated signaling may restore physiological fibrolysis while avoiding the limitations of global protease inhibition.
2. Targeting the cellular–matrix interface: Integrins and related adhesion receptors regulate recruitment, retention, mechanotransduction, and activation. Their selective modulation may simultaneously interrupt several pro‐fibrotic processes.
3. Target signaling hubs rather than isolated downstream mediators such as integrin–TGF‐*β*, GARP–TGF‐*β*, integrin–PAK–YAP, SMAD6/NF‐*κ*B, and other highly connected pathways, may offer greater therapeutic leverage while preserving essential physiological signaling.

These principles converge on a common therapeutic goal of converting the fibrotic niche from a self‐sustaining state of pathological wound healing toward a state of active resolution.

## 7 Limitations and Open Questions

Despite the rapid progress in spatial biology and matrix‐focused fibrosis research, several limitations remain. First, many candidate targets identified by single‐cell or spatial approaches require functional validation to distinguish causal drivers from markers of advanced diseases. Second, the cellular identity of fibrocytes and related circulating stromal or myeloid populations in human tissue remains difficult to define because of phenotypic overlap with inflammatory monocytes and macrophage‐like states. Third, many anti‐fibrotic pathways are context‐dependent and may have different effects during active injury, established fibrosis, and regression. Finally, liver fibrosis often coexists with metabolic dysfunction, inflammation, vascular remodeling, and a risk of carcinogenesis, indicating that antifibrotic therapy will probably need to be combined with interventions targeting the underlying disease driver.

These challenges do not weaken the rationale for precision antifibrotic therapy, but emphasize the need for mechanism‐guided validation. Future studies should determine which spatial niches are conserved across patients, which molecular circuits are disease‐specific, and which biomarkers can identify patients who are most likely to respond to a given intervention. In this sense, the central task is not only to expand the list of antifibrotic targets, but also to define the biological context in which each target becomes therapeutically active.

## 8 Conclusion

The next generation of antifibrotic therapies are unlikely to emerge from a single universal inhibitor of collagen synthesis. Instead, it depends on the ability to identify and selectively modulate the molecular nodes that maintain pathological communication between immune cells, stromal cells, and the ECM. Our work on MMP/TIMP biology and fibrocyte‐mediated matrix remodeling, together with broader contributions to the molecular and cellular understanding of hepatic fibrosis, support this shift from structural inhibition toward functional reprogramming. Fibrocytes are particularly useful in this regard. Their limited contribution to collagen production contrasts with their substantial regulatory influence, positioning them as potential network nodes rather than conventional therapeutic targets. Their effects on MMP3/MMP13, inflammatory mediators, and SMAD6 further illustrate how immune–stromal communication can converge on matrix remodeling and intracellular signaling.

Therefore, the therapeutic opportunity lies in selectively modulating matrix‐remodeling enzymes, adhesion receptors, and signaling hubs according to the molecular architecture of an individual patient's fibrotic niche. Spatial multiomics and functional perturbation are crucial for identifying these actionable states. Such an approach could transform antifibrotic therapy from a broadly suppressive treatment to a mechanism‐guided strategy that restores matrix homeostasis, interrupts pathological mechanotransduction, and re‐establishes the capacity of the liver to resolve fibrosis. Consequently, the central therapeutic question should no longer be: How can collagen production be inhibited? However, which molecular nodes sustain pathological matrix remodeling and fibro‐inflammatory signaling in a given patient and can they be selectively reprogrammed to restore fibrosis resolution?

The future of antifibrotic therapy lies in the transition from pathway inhibition toward network reprogramming. The hepatic fibrotic niche is maintained by reciprocal interactions between matrix architecture, adhesion receptors, mechanical forces, inflammatory signals, and intracellular transcriptional programs. Matrix remodeling enzymes such as MMPs and TIMPs regulate the physical and signaling properties of the ECM, integrins translate these extracellular cues into intracellular responses, and signaling hubs including TGF‐*β*/SMAD, FAK–RhoA/ROCK, and YAP/TAZ consolidate these inputs into persistent cellular states. Recent spatial and single‐cell studies have provided a means of resolving these mechanisms at cellular and anatomical resolutions. The resulting opportunity is not simply to discover more antifibrotic targets, but also to determine which target is functionally dominant in which cellular state, in which spatial niche, and at which stage of disease [58].

This is the essence of precise antifibrotic therapy. Rather than globally suppressing fibrosis, treatment should selectively dismantle the molecular architecture that prevents its resolution, restoring productive matrix turnover, interrupting pathological cell–matrix adhesion and mechanotransduction, and reprogramming signaling hubs that sustain myofibroblast activation. In this framework, the goal is not to completely eradicate the scar‐forming response. This is the restoration of matrix homeostasis and tissue competence for the resolution of fibrosis. The combination of spatial multi‐omics, functional perturbation, and mechanism‐guided drug development may therefore allow antifibrotic therapy to evolve from empiric pathway inhibition toward genuinely precision‐based, niche‐directed, and resolution‐oriented therapeutic strategies.

## Author Contributions

E.R. and R.W. made substantial contributions to the conception and design of this article and performed the data analysis and interpretation.

## Funding

The authors acknowledge support by grants from the German Research Foundation, DFG (Grant No.s RO957/13‐1 and RO3714/4‐2), the von‐Behring‐Röntgen Foundation (Grant No. 73‐00001), and the Deutsche Krebshilfe (Grant No. 70115581).

## Ethics Statement

The authors have nothing to report.

## Conflicts of Interest

R.W. is the Executive Editor of the *Journal of Cell Communication and Signaling* and Editor of the Special Issue “Cell‐Cell and Extracellular Matrix Signaling in Multiorgan Fibrosis: Mechanisms, Quantitative Therapeutic Thresholds, and Translational Targeting for Tissue Remodeling.” However, he was not involved in any step of the editorial process, notably, reviewer selection, manuscript handling, and decision‐making. E.R. received honoraria for lectures and consultancy services, as well as reimbursement of travel and accommodation expenses, from the following companies: Abbvie, BMS, Boehringer Ingelheim, Falk Foundation, Gilead, Ipsen, Madrigal, Medac, MSD, Norgine, Pfizer, PM Results, Streamed up, Takeda, Thieme, Wiley Verlag.

## Data Availability

No new data were generated in this study.
